# When Gesture “Takes Over”: Speech-Embedded Nonverbal Depictions in Multimodal Interaction

**DOI:** 10.3389/fpsyg.2020.552533

**Published:** 2021-02-11

**Authors:** Hui-Chieh Hsu, Geert Brône, Kurt Feyaerts

**Affiliations:** Department of Linguistics, Faculty of Arts, University of Leuven, Leuven, Belgium

**Keywords:** depiction, multimodality, gesture, iconicity, embedding

## Abstract

The framework of depicting put forward by Clark (2016) offers a schematic vantage point from which to examine iconic language use. Confronting the framework with empirical data, we consider some of its key theoretical notions. Crucially, by reconceptualizing the typology of depictions, we identify an overlooked domain in the literature: “speech-embedded nonverbal depictions,” namely cases where meaning is communicated iconically, nonverbally, and without simultaneously co-occurring speech. In addition to contextualizing the phenomenon in relation to existing research, we demonstrate, with examples from American TV talk shows, how such depictions function in real-life language use, offering a brief sketch of their complexities and arguing also for their theoretical significance.

## Introduction

We communicate meaning to each other in different ways: by creating a physical analog, by relating ourselves to the physical world, or by assigning a sign to the meaning (Peirce, 1932; Clark, 1996; see also Goodman, 1968). The communication is in turn carried out in different channels: through speech, through nonverbal channels — such as manual gesture, eye gaze, and vocalization — or through a combination of multiple different channels. Linguists have long focused on how meaning is communicated through speech, primarily when it comes to the use of signs symbolically imposed on meanings, but also where the speaker makes meaning by anchoring themselves to the environment. With the multimodal turn in linguistics, as well as the resulting revitalization of interests in iconicity (Jakobson, 1966; Haiman, 1983; Simone, 1995; Wilcox, 2004; Perniss et al., 2010; Mittelberg, 2014), researchers have also examined how the speaker employs nonverbal signals alongside speech, such as co-speech iconic gestures (McNeill, 1992; Kendon, 2004; Cienki and Müller, 2008; Streeck, 2009) and pointing that accompanies verbal indices (Clark, 2003; Goodwin, 2003; Kita, 2003; Mondada, 2014; Langacker, 2016; Ruth-Hirrel and Wilcox, 2018). The recognition of signed languages as full-fledged linguistic systems likewise prompted curiosity about how nonverbal signals are used and coordinated to carry out the functions spoken languages serve (e.g., Stokoe, 1960; see also Vermeerbergen, 2006; Müller, 2018). While the current state of the art is a long way from the near-exclusive focus on symbolic signs at the onset of modern linguistics, the puzzle is not complete. Among the missing pieces are cases where meaning is communicated through iconic, nonverbal signals, in the absence of simultaneously co-occurring speech. Having only been explored in a handful of studies, this topic remains largely uncharted territory in the linguistics literature.

To contextualize, as well as better understand, phenomena that fall within this overlooked domain, we turned to the framework of language use proposed by Clark (1996) as a starting point. Building on Peirce’s (1932) trichotomy of signs — icons, indices, and symbols — Clark distinguishes three methods in which meaning is signaled in language — depicting, indicating, and describing (as) — contextualizing the semiotic triangle in present-day linguistics. In a recent paper, Clark (2016) proposes the theoretical framework of the staging theory, in which he further elaborates on depicting as a basic method of communication. With examples from empirical data, he shows how depictions are employed in interaction to serve communicative functions, singles out numerous analytical dimensions that may prove crucial for the understanding of depictions, and, importantly, argues for the relevance of depicting to the study of language use that is on a par with indicating and describing.

Specifically, Clark (2016, pp. 324–325) defines depictions as iconic physical scenes people create and display, with a single set of actions at a single place and time, for the addressee to use in imagining the scenes depicted. They are physical analogs people produce, for the purpose of communicating meanings to which the analogs bear perceptual resemblance. Given the array of articulators the speaker is equipped with, depictions draw on various resources across different modalities, including manual gesture, bodily posture, head movement, facial expression, eye gaze, onomatopoeia, vocalization (Clark, 2019), any other “visible bodily action” (Kendon, 2004) — or even more broadly, any other “publicly intelligible action” (Mondada, 2019).

### Depiction in Interaction

Found in an episode of *The Tonight Show Starring Jimmy Fallon*, the following example illustrates what a typical depiction in real-life language use looks like. In the excerpt, Kaley Cuoco, the talk show guest, recounts her experience of doing a ‘‘canyon swing.’’^1^

(1) Kaley Cuoco explains what canyon swing is: “… and you’re supposed to just walk off, and it’s a six-second free fall, and (*swings right arm back and forth, parallel to frontal plane*)^a^ then you swing, for ten minutes.’’^2^

— *The Tonight Show Starring Jimmy Fallon*^3^

**FIGURE 1:**
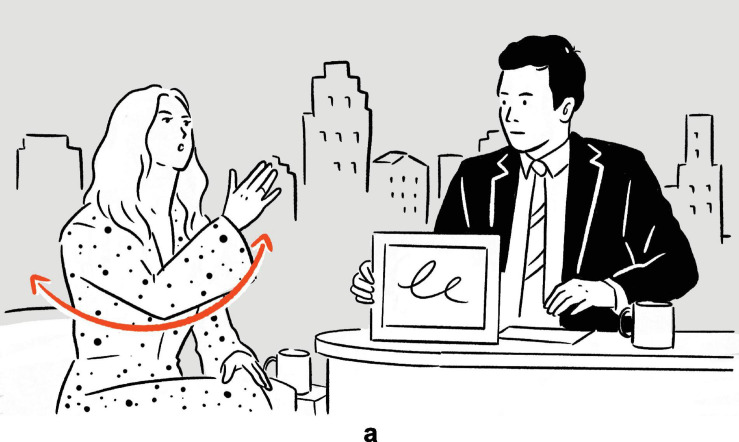
Depiction in (1).

In addition to verbally describing the event of swinging by uttering *you swing*, Cuoco also stages a depiction simultaneously. Specifically, she deploys and coordinates a set of actions, consisting among others of a pendulum-like movement of her entire right arm. In a highly schematic fashion, the actions abstract from the depicted event of the actual canyon swing as she experienced it, to which the actions are iconic, with Cuoco’s right arm being mapped onto the string of the canyon swing, and her right hand modeling her own body on the canyon swing.^4^ The result is a depiction which, within the interpretive framework (see e.g., Bloom, 2010) that is the local context of language use in the exchange between Cuoco and Fallon, manifests physical resemblance to the depicted scene of the canyon swing.

By creating and displaying the depiction, Cuoco provides her audience — in this case Fallon, the audience in the recording studio, and the audience of the show as broadcast — with rich semiotic resources with which to imagine and comprehend the canyon swing scene, in a way that is concrete and perceptually tangible: Normally, with the descriptive verbal phrase *you swing* alone, the audience imagines the swinging based primarily on the symbolic (and therefore largely arbitrary) form-meaning relation associated with the verbal phrase. With the aid of the depiction, the audience is afforded additional semiotic resources with which to imagine and therefore understand the swinging — including the manner, direction, and speed — in a more direct, albeit highly schematic, manner, as the link between the form of the depiction and its meaning is iconically motivated.

### A Schematic Vantage Point

Essentially, depictions, as defined by Clark within the staging theory, make up cases of language use where the relation between the semiotic signal and its denotation is iconic, contrasting with descriptions — whose form-function relation is symbolic — and indications — whose form-function relation is indexical (Peirce, 1932; Clark, 1996, 2016). Importantly, the three ways in which meaning can be signaled are not mutually exclusive. It is possible, and indeed often the case, for a communicative form to signal meaning in more than one way: A depiction of someone finger-pointing at something, for example, is both depictive and indicative; conventionalized ideophones such as *meow* and *whack* are descriptive as well as depictive (Dingemanse, 2017a); likewise, depicting constructions in signed languages exhibit properties associated with both descriptions and depictions (Ferrara and Hodge, 2018). Depicting, indicating, and describing are therefore better considered, not as discrete categorical notions, but as properties or dimensions of communicative signals, a view that finds advocates in more recent studies (McNeill, 2005; Mittelberg and Evola, 2014). In this sense, a prototypical depiction is really a communicative signal whose depictive property is more salient than its indicative and descriptive properties.

While Clark’s approach to depictions may be new to the field, the notion of depicting itself is not, and neither are the plethora of phenomena that fall within Clark’s definition of depicting (though many of these have been marginalized in the literature, see subsection “An imbalance in the Literature” and Dingemanse, 2017b). The very term of depicting has been used by a great number of researchers — some in more clearly delimited senses than others — to refer to various different subsets of iconic, nonverbal strategies of communication. Examples of a more systematic use of the term depicting can be found in the research of Müller and Streeck. Drawing on an analogy to the techniques employed by visual artists, Müller (1998b) identifies four basic techniques of gestural depiction: acting, molding, drawing, and representing, later breaking them down into two: acting and representing (Müller, 2014). Observing how a single object can be depicted by the hand in multiple different ways, she explores the interplay between gestural representation and conceptualization. Streeck (2009), on the other hand, views gestures as organic products of humans acting in the material world as well as in interaction with each other. Examining empirical data in a “micro-ethnographic” fashion, he identifies, heuristically, a dozen depiction methods, positing that “to depict a phenomenon means to analyze and represent it in the terms that the given medium, communicative modality, or symbol system provides” (Streeck, 2008, p. 286).

Also covered by Clark’s notion of depicting are manual gestures with an iconic form-meaning relation, a topic that has been explored by a great number of researchers, though not necessarily using the term depicting (e.g., Calbris, 1990; McNeill, 1992, 2005; Gullberg, 1998; Kendon, 2004; Sowa, 2006; Cienki and Müller, 2008). Depending whether the denotation is something concrete in the material world, these gestures are often divided into two separate groups: “iconic” (or “representational,” “referential,” “imagistic”) and “metaphoric” (or “conceptual,” “ceiving”) (see the reviews in Kendon, 2004; Streeck, 2008; Mittelberg and Evola, 2014). On a more schematic level, Clark’s notion of depicting covers also phenomena which are often approached separately and independently, but which share the same defining property of iconicity. These include topics such as quotation, demonstration, enactment, pantomime, mimesis, facial gesture, ideophone, constructed action, and depicting construction (e.g., Mandel, 1977; Clark and Gerrig, 1990; Chovil, 1991; McNeill, 1992; Wade and Clark, 1993; Kita, 1997; Gullberg, 1998; Liddell, 2003; Kendon, 2004; Zlatev, 2005; Taylor, 2007; Cienki and Müller, 2008; Vandelanotte, 2009; Cormier et al., 2012; Dingemanse, 2013; Cormier et al., 2016; Gärdenfors, 2017). Furthermore, depictive semiotic signals are ubiquitous not just across said topics, but are prevalent across modalities, and observed across communicative ecologies (such as hearing to hearing, and deaf to deaf; see Ferrara and Hodge, 2018).

As the above overview shows, phenomena in language use that pertain to iconicity have been explored by many, from various perspectives and in different theoretical frameworks. For the present study, Clark’s (2016) account of depicting was chosen as the starting point through which to explore the aforementioned oversight in the literature, for the reason that Clark’s definition of depicting is a well delimited one, but more importantly, because it provides a schematic vantage point from which to approach iconicity. Rather than a mere change of terminology, the framework situates existing research in a bigger picture, uniting and consolidating numerous research traditions. This affords the researcher the possibility of observing iconic language use on a more schematic level, and, in turn, the potential to identify patterns that have hitherto eluded scholarly attention. Indeed, some early findings using Clark’s framework of depicting have already been reported, for both spoken and signed language interactions (e.g., Ferrara and Hodge, 2018; Hodge et al., 2019; Hsu et al., to appear). Given that this framework was put forward only fairly recently, it is reasonable to expect more fine-grained analyses in the near future.

### The Present Study

In the following sections, we start from one of the central theoretical distinctions in Clark’s framework, namely the four-way typology of depictions (section “Clark’s Typology of Depictions”). A closer examination reveals potential insufficiencies of the typology, leading to problems in categorization. In light of this, we tap into a corpus of American TV talk shows that we constructed specifically for this purpose (section “Methods”). Through systematic data annotation and operationalization of relevant theoretical notions in Clark’s framework, we establish a methodology for researching depictions that is both empirically grounded and theoretically valid. Confronting Clark’s proposed typology with real-life usage events taken from the corpus (section “Depiction Type Attribution”), we zero in on the issues encountered in depiction type attribution, pinpointing underspecification and form-function conflation as the major underlying causes. This critical examination further leads to an alternative, gradient conceptualization of Clark’s original typology. Crucially, this alternative conceptualization brings the aforementioned overlooked domain to the fore, namely cases where meaning is communicated iconically, nonverbally, and without simultaneously co-occurring speech. A review of existing studies then reveals a curious imbalance in the literature, between gesture employed with and without co-occurring speech: Ubiquitous in language use, cases of gesture without temporally overlapping speech have been widely acknowledged by researchers, but unlike those with cotemporal speech, they have not received proportionate scholarly attention.

In view of this, we zoom in on a subset of phenomena within this marginalized domain: “speech-embedded nonverbal depictions” — which we define in more technical terms as “depictions that are embedded in speech, but that are not depictions of non-depictive speech” (section “Speech-Embedded Nonverbal Depictions”). This definition excludes depictions of descriptive and indicative speech (e.g., canonical quotations), but takes into account cases of depictive speech (e.g., ideophones), allowing us to focus on depictions that have until recently been largely overlooked. Taking embedding in a strictly formal sense — in terms of temporal overlap — this approach also steers clears of the problems of Clark’s original typology. Following a delimitation of speech-embedded nonverbal depictions, a brief sketch is offered of how such depictions function in naturally occurring discourse. With examples from the TV talk show corpus, we demonstrate the theoretical significance of speech-embedded nonverbal depictions in relation to current topics in the literature of relevant fields of inquiry, calling for further research on this marginalized topic along various dimensions.

Essentially, the aim of the present paper is first and foremost to establish a case for speech-embedded nonverbal depictions, by demonstrating their theoretical significance, but also by laying out the methodological groundwork for systematic empirical investigations. The findings — methodological, theoretical, and empirical — of the present study are therefore reported throughout sections “Methods,” “Depiction Type Attribution,” and “Speech-Embedded Nonverbal Depictions,” although the more technical discussions of empirical tokens are concentrated in section “Speech-Embedded Nonverbal Depictions.”

As the term “speech-embedded nonverbal depiction” suggests, the main argument of the present study builds in part on distinctions such as “verbal vs. nonverbal.” The use of such dichotomous terms calls for clarification. On the technical level, modality and signaling method need to be teased apart. Like other modalities, speech can be depictive, indicative, descriptive, or any combination thereof. Since the focus here is on depicting, unless otherwise specified, we use “speech,” as well as related terms and modifiers such as “verbal” and “utterance,” as a shorthand term for non-depictive speech, that is descriptive or indicative speech, where speech is understood in a modality-agnostic (Dingemanse, 2019) sense compatible with both spoken and signed languages. The distinction is therefore really between different combinations of signaling method and modality (e.g., “non-depictive speech vs. depictive signals”), and not between different modalities. The specific use of the terms serves the purpose of naming, and therefore tackling, the specific phenomena in question, rather than asserting rigid categorical boundaries based on a dichotomy between “language proper” and “paralinguistic noise” such as gesture. In line with most researchers in relevant fields of inquiry, we view all communicative behavior that is deemed (intentionally) meaningful (see Kendon, 2004) as integral to talk, to speaking, and to language use. It follows that the seemingly discrete categorical notions are really heuristics that guide the recognition of phenomena along the messy and overlapping continua that constitute language use. As Streeck (2009, p. 11) also acknowledges, categorization “helps us organize our analysis, […] reminds us of the wide range of different uses to which gesture is put, and thus keeps us from drawing overly broad generalizations from a narrow data-set.”

## Clark’s Typology of Depictions

Based on the functional relations between depictions and their adjacent or accompanying (non-depictive) speech, Clark (2016) puts forward a typology consisting of four types of depictions: adjunct (where the depiction, acting like a nonrestrictive modifier, co-occurs with and illustrates descriptive speech), indexed (where the depiction is indexed by an indexical device in speech), embedded (where the depiction takes up a syntactic slot in a descriptive verbal utterance), and independent (where the depiction stands alone). Cuoco’s depiction in (1), repeated in (2), is an example of an adjunct depiction, as her depiction co-occurs with, and illustrates, the descriptive verbal phrase *then you swing*, thereby adding to it iconic imagistic details of the event, such as the physical configuration of the swing, and the manner and directionality of the movement, though only schematically. The depictions in (3)–(5), which are manipulated variations based on (2), illustrate the other three types of depictions in Clark’s typology.

(2) Adjunct depiction: “… and you’re supposed to just walk off, and it’s a six-second free fall, and (*swings right arm back and forth, parallel to frontal plane*) then you swing, for ten minutes.”
(3) Indexed depiction: “… and you’re supposed to just walk off, and it’s a six-second free fall, and then you swing like (*swings right arm back and forth, parallel to frontal plane*) this, for ten minutes.”
(4) Embedded depiction: “… and you’re supposed to just walk off, and it’s a six-second free fall, and then you [*swings right arm back and forth, parallel to frontal plane*], for ten minutes.”
(5) Independent depiction: Jimmy Fallon: “How does a canyon swing work?” Kaley Cuoco: “[*swings right arm back and forth, parallel to frontal plane*]”

As the depiction in (3) is connected to the rest of the utterance by the verbal demonstrative *this*, it is categorized as an indexed depiction (but see subsection “Embedding”). Importantly, indexed depictions differ from most deictic expressions, in that the referent of the indexical device is not something that already exists (either physically or conceptually), but the depiction created by the speaker for local purposes. In (4), the depiction is embedded in speech, in the sense that it fills the syntactic slot where a verbal phrase (e.g., *swing in the canyon*) otherwise would; it is therefore an embedded depiction. In (5), Cuoco answers Fallon’s question not with descriptive speech, but with a set of nonverbal, depictive actions, which stands alone and contributes to the discourse independently, hence an independent depiction.

The categorization might, at first sight, appear to nicely capture the various possible functional relations between depictions and speech; however, it really only is the case with tokens that are prototypical exemplifiers of the four depiction types. Upon careful consideration, ambivalence surfaces, in gray areas where the categories overlap. For instance, if the depiction in (2) did not co-occur with, but followed the phrase it illustrates (*then you swing*), would it still count as an adjunct depiction, and not as an independent one? Is an embedded depiction that takes up a syntactic slot on the clausal (or even sentential) level still to be categorized as an embedded depiction, and not as an independent one? Such issues only become exacerbated once the typology is confronted with the messy, heterogeneous tokens in empirical data.

While Clark’s typology is likely put forward, not as a definitive assertion about discrete categories, but in a heuristic way in which to demonstrate the diverse depiction-speech relations, it was taken as the starting point for our empirical investigation on depicting. In what follows, we critically scrutinize the four depiction types as defined by Clark in a bottom-up fashion, comparing them to the empirically attested phenomena observed in a corpus constructed for this purpose — a process through which previously overlooked issues can be identified and addressed, potentially leading to an understanding of depicting that is more well-rounded, both theoretically and empirically.

## Methods

The data examined for the present study comprise video recordings of American TV talk shows, which were chosen for a number of reasons: While the topics of the talk show episodes may be predetermined, there is nonetheless a high level of spontaneity in the way the topics are actually delivered or discussed by the hosts and guests. Video recordings of talk shows are plentiful, easy to collect, and come in good quality. In addition, the unbalanced interpersonal power dynamics found in certain settings (e.g., in contexts of instruction, see Goldin-Meadow, 2003; Hsu et al., to appear) are to a large extent absent. Indeed, a growing number of studies on multimodal communication have examined television data for similar reasons [see the studies drawing on the databases of the Red Hen Lab (www.redhenlab.org) and the TV News Archive (archive.org/details/tv); e.g., Steen and Turner, 2013; Winter et al., 2013; Zima, 2017; Hinnell, 2018, 2019]. On top of all these, TV talk shows abound in recounts of past experiences and enactments of hypothetical scenarios, both of which contribute to the richness of depicting.

### Corpus and Annotation

To avoid generalization over individual idiosyncrasies, a corpus of video recordings was constructed, comprising video clips randomly retrieved from the official YouTube channels of four American TV talk shows: *The Ellen DeGeneres Show*, *Late Night with Seth Meyers*, *Conan*, and *The Tonight Show Starring Jimmy Fallon*. Specifically, we examined only segments of host-guest interaction — that is, where the host and guest(s) are both physically present in the recording studio, and where they interact with one another — as these segments approximate canonical, spontaneous face-to-face interaction more so than other types of “interaction” on TV (e.g., where the host speaks directly into the camera, see Turner, 2017). In total, 147 video clips were examined, amounting to a total duration of approximately 10 h 37 min.

The video data were imported into ELAN,^5^ where tokens of depictions were identified and segmented (see subsection “Unit of Analysis” for segmentation). Aware of the problems of form-function conflation such as circularity (see e.g., Croft, 2001), a strict line was drawn between the formal and functional properties of depictions. Given the complexity and heterogeneity of depictions, for each of the tokens, we describe the salient form features (features of “articulator form,” rather than “gesture form”; see Hassemer, 2016) of the actions that are core to the depiction (the “modality-agnostic stroke”; see subsection “Unit of Analysis”). Specifically, McNeill’s (1992) gesture space is referenced in the description of location. For other parameters such as articulator shape, movement, and orientation, the annotation is informed by Bressem’s (2013) form-based annotation scheme for manual gestures, which also steers clear of any functional interpretations. On top of the description of depictions per se, our annotation also includes, among others, depiction type (in Clark’s typology), immediately adjacent or overlapping speech, grammatical level of embedding, as well as parameters pertaining to depiction-speech relations. A screenshot of our full annotation in ELAN (which includes annotation tiers beyond the scope of the present paper) can be found in the Appendix. In total, 217 tokens of our target phenomenon — speech-embedded nonverbal depictions — were identified and annotated in our corpus (see section “Speech-Embedded Nonverbal Depictions” for a full definition of such depictions), providing the empirical basis of the present study.

Due to the limitations of format — in the sense that it is not possible to include dozens of video clips with sufficient length to cover all relevant contextual information — the descriptions of the tokens in the present paper were adapted accordingly, to facilitate the reader’s understanding and imagination of the actions described. In addition to overly trivial details being omitted, functional descriptions are supplemented where a purely form-based description would be overly lengthy and confusing. These functional descriptions are always marked and preceded by the phrase *as if*.

### Unit of Analysis

The construction of our corpus, specifically our attempt at systematic annotation, was not without challenges. Among them is the lack of a readily operationalizable unit of depiction. In his account of depicting, Clark (2016) does not spell out the segmentation of depiction tokens (neither does Müller, 2014 or Streeck, 2008), which is essential to the establishment of the basic unit of analysis, and therefore to systematic annotation. While clear-cut examples such as (1) do exist, more often than not, a depiction is preceded or followed, with or without speech “intervening,” by another depiction, which can be either similar or distinct in form and meaning, as illustrated in the following examples. For clarity, they are presented with our final segmentation, explained immediately below.

(6) Lauren Ambrose on backstage costume change on Broadway: “I mean sometimes it’s like twenty seconds, for like, full-on, [*vocalizes whistle-like* fsss *sound, moves both hands vertically, fingers spread, in opposite directions, in front of head and torso*]^a^ — [*vocalizes whistle-like* ffft *sound, gazes at the front, into the distance, moves both hands along sagittal axis away from body, fingers spread, palms away from body*]^b^.”

— *Late Night with Seth Meyers*

**FIGURE 2:**
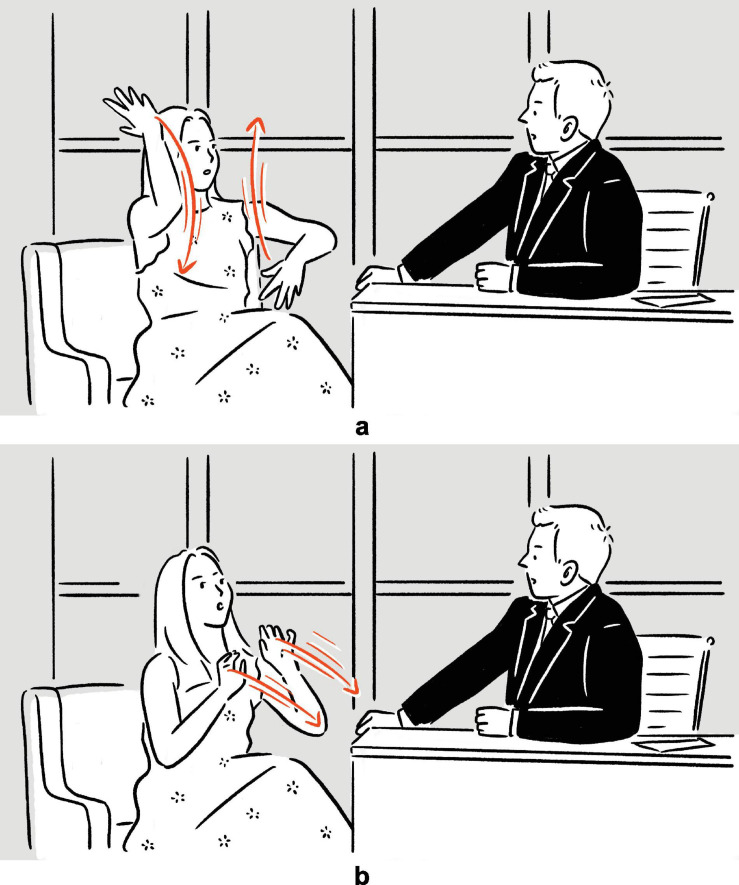
Depictions in (6).

(7) Tracy Morgan explains what bingo wing is: ‘‘When an old woman hits bingo, she goes, [*vocalizes* bingo,^6^ *raises and shakes both arms, elbows bent*]^a^, and then [*raises and shakes left arm, left elbow bent; moves right hand, fingers spread, back and forth under and perpendicular to left arm*]^b^, bingo wings, [*raises and shakes both arms, elbows bent*]^c^ (.) [*raises and shakes both arms, elbows bent*]^d^.’’^7^

— Conan

**FIGURE 3:**
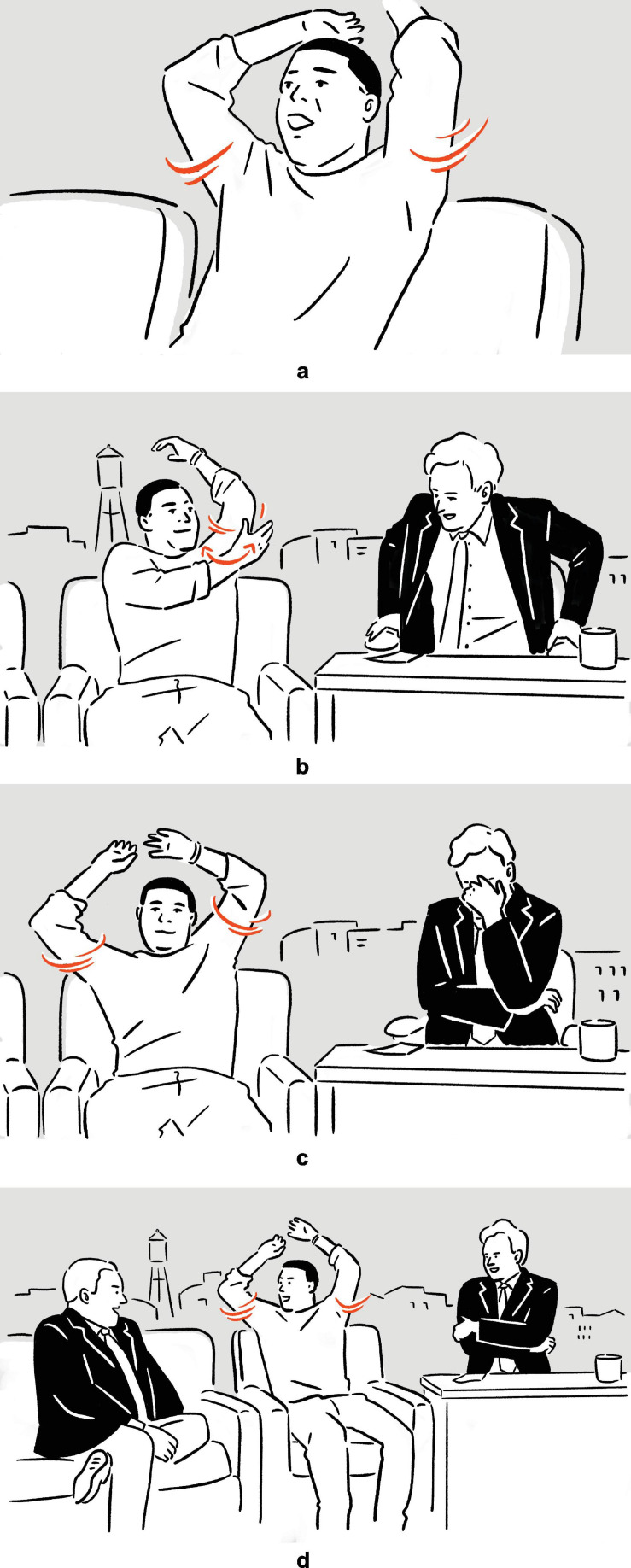
Depictions in (7).

In (6), Ambrose recounts her experience of performing on Broadway, specifically the backstage costume change operations which she finds unbelievably fast. In the first set of actions, with her moving hands standing for the hands of the multiple members of backstage personnel quickly working on her clothes and makeup, and with the rest of her body portraying herself in the depicted scene, the highly efficient change of costumes is depicted^8^; in the second set, she depicts someone already pushing her, with their hands, back to the stage. The two sets of actions are deployed consecutively, without a pause. They utilize the same channels of communication (mainly, vocalization and manual movements), but the actions are distinct in form (*fsss* vs. *ffft*, vertical movement vs. movement along the sagittal axis). At the same time, the two sets of actions are functionally interrelated, as they each depict a part of a larger sequence of events. In (7), Morgan explains the (folk) etymology and concept of “bingo wings” — the flabby triceps area that wobbles as the arm moves — through four sets of actions, which involve him shaking his own arm in an exaggerated manner so as to make the triceps shake, whilst vocalizing *bingo*. Here, the four sets of actions are “interrupted” by speech and a pause, but they are very similar in both form and meaning. In fact, all except for the second set are essentially iterations of the same actions. These two tokens exemplify the commonly observed mismatch in terms of sequentiality, form, and meaning — sequentially consecutive depictions, for instance, can be distinct in form but interrelated in meaning, while depictions that are separated by descriptive words can be identical to each other both in form and meaning — posing challenges to systematic segmentation.

While there is probably no universally valid definition of a unit of depiction, to ensure consistency in annotation, we adapted and operationalized the notion of the gesture phrase as the basic unit of depiction for the present study. The gesture phrase, as defined by Kendon (1972, 1980) primarily for the study of manual gesture, consists of the preparation phase, the stroke, and any subsequent sustained position. Given the fact that depictions often make use of modalities other than manual gesture, we schematized from Kendon’s definition, making the gesture phrase a modality-general — or, following Dingemanse (2019), “modality-agnostic” — notion, where the stroke can be carried out by any possible articulator, or combination of articulators. In this sense, a unit of depiction consists of a stroke of action (be it manual gesture, vocalization, head tilt, leg or torso movement, etc.) as its core, with its start marked by the onset of the preparation phase of the action, its end either by a complete rest, or by the onset of another modality-agnostic gesture phrase. The operationalization of the gesture phrase as a modality-agnostic notion is in line with recent works on comparable topics (e.g., Ferrara and Hodge, 2018; Dingemanse, 2019), but also motivated by Kendon’s (2004) view of gesture as “visible bodily action,” as well as Mondada’s (2019) notion of “publicly intelligible action.” Adopting a broader sense of the term “gesture” that is not limited to manual actions, we take into consideration all nonverbal signals that contribute to the meaningfulness of the depictions in question, including visual but also auditory ones.

It is in this way that the depictions in (6) and (7) were segmented, as indicated by the brackets above. Despite the two depictions in (6) being staged back to back, and despite their shared semantic thread, they exhibit two distinct sets of actions, with two distinct strokes of actions (both of which with simultaneous utilization of vocalization and manual gesture), rendering them not one but two units of depiction. In (7), the four sets of actions share many common features, with the fourth being a reiteration of the third. However, since each of them is followed by either a complete rest or another gesture phrase, they make up four gesture phrases, and therefore four units of depiction in our annotation. All other tokens in our corpus were segmented following the same principle.

## Depiction Type Attribution

Confronted with our TV talk show data, Clark’s staging theory indeed captures much of the complexities of depictions rather intuitively and coherently, especially in the identification of depictive properties in communicative signals. At the same time, however, this process also revealed potential insufficiencies. In addition to methodological issues such as segmentation, also foregrounded are problems on a more theoretical level, including the aforementioned issue of depiction type attribution.

As mentioned in section “Clark’s Typology of Depictions,” Clark’s (2016) definition of depiction types leaves gray areas for non-prototypical cases. This is confirmed by our attempt at imposing the typology on our corpus data. Some of the frequently encountered challenges are illustrated by the following example.

(8) Tracy Morgan on the quality of his facial muscles: ‘‘Yeah, I’m your rubber-band man, [*vocalizes* brbrbrbr *sound,^9^ shakes head sideways quickly, causing facial muscles to vibrate accordingly*]^a^.’’^10^

— Conan

**FIGURE 4:**
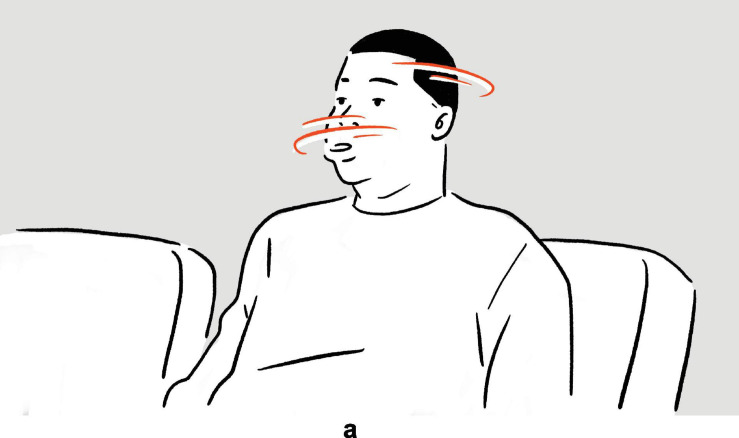
Depiction in (8).

Following the verbal phrase *rubber-band man*, Morgan depicts the elastic, rubber-like quality of his skin, by shaking his head violently so that the cheeks wobble, thereby illustrating, metonymically, the verbal phrase.

As defined by Clark (2016, p. 326), adjunct depictions are the ones that are “timed to overlap with” their verbal affiliates to which they are adjoined, so as to elaborate on them “as if they were non-restrictive relative clauses” or nonrestrictive modifiers. In (8), as the brackets indicate, there is no temporal overlap between the speech and the depiction, although at the same time, the depiction elaborates on its verbal affiliate *rubber-band man*, albeit metonymically, rendering it unclear whether it is an adjunct depiction. If, for the sake of discussion, we do not categorize it as an adjunct depiction, for the reason that it does not share all the properties of a prototypical adjunct depiction, further issues arise: Does it belong to embedded depictions, which “function as parts of utterances — as if they were words, phrases, or other segments,” or to independent depictions, which make “independent contributions to the discourse” (Clark, 2016, pp. 325–326)? In other words, is the depiction embedded in the verbal sentence as if it were an apposition, therefore a part of the utterance, or does it make a contribution that is independent of the preceding utterance (but see subsection “Embedding” for the issue of independence; see also Lehmann, 1988; Hodge and Johnston, 2014 on comparable phenomena observed in other communicative ecologies)? On top of that, how “independent” is “independent” enough? What kind of independence is at issue: syntactic, semantic, or something else? These questions suggest calibration may be needed before the typology can be applied empirically.

Indeed, a critical review of the typology brings to light two major causes of confusion: underspecification and form-function conflation. Underspecification is most manifest where independent and embedded depictions are concerned — it is unclear what level, and what kind, of independence is sufficient for a depiction to be categorized as “independent.” Similarly, for indexed depictions, it is not specified whether they include only depictions indexed by indexical pronouns (e.g., *this* in *I’d do it like this*), or also those indexed by indexical modifiers (e.g., *that* in *they chose that color*), despite the fact that indexical pronouns and indexical modifiers are indexical in distinct ways (see subsection “Embedding”).

Form-function conflation, on the other hand, is a problem that is inherent in the typology itself. Although the four types of depictions are, as Clark puts explicitly, defined in terms of their discourse functions, both formal and functional criteria are present in their definition. Take the aforementioned case of adjunct depictions. While it is indeed a functional definition that an adjunct depiction elaborates on its verbal affiliate in a way that is similar to a non-restrictive relative clause, the criterion that an adjunct depiction is “timed to overlap with” (Clark, 2016, p. 326) its affiliate is unequivocally a formal one. Conflation is also found among different functional notions. For example, as indexation and embedding are not two mutually exclusive functional concepts, ambiguity often surfaces where an indexed depiction is itself part of an embedded depiction. In fact, mutual inclusion can, strictly speaking, be found among all of the canonical functions associated with each of the depiction types — elaboration, indexation, embedding, and independent meaning contribution. Issues such as these call for thorough reconsideration of depiction categorization in relation to speech.

### Typology of Depictions Reconceptualized

**FIGURE 5:**
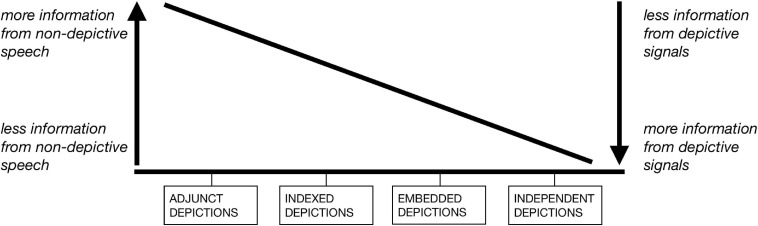
Continuum of information contribution from non-depictive speech and depictive signals.

Serving as the starting point for the present study, the critical examination of Clark’s (2016) typology presents, more importantly, an analytical process toward a better understanding of depictions. Among the results of this process is a reconceptualization of the depiction types, which in fact foregrounds some of the implicit insights of Clark’s original typology. In this subsection and the next, we consider the theoretical implications of this reconceptualization, visualized in Figure 5, before tackling the issues of the typology raised above.

The four depiction types are placed along a continuum, with varying levels of information contribution from two different combinations of modality and signaling method: non-depictive speech (i.e., indicative and descriptive speech) and depictive signals (e.g., depictive manual gesture, depictive bodily movement, depictive speech), where speech is understood in the above-mentioned modality-agnostic sense.^11^ On the left half of the continuum are cases where more information is communicated through non-depictive speech, and where relatively less information comes from depictive signals. Here we find adjunct and indexed depictions: As adjunct depictions illustrate what is said in the descriptive speech they co-occur with, part of the composite meaning is conveyed through their co-occurring speech [cf. “composite utterance” (Enfield, 2009) and “multimodal attribution” (Fricke, 2008, cited in Bressem, 2014; Ladewig, 2020)]. In the case of indexed depictions, indicative speech provides essential deictic information, directing the addressee’s attention toward the depiction, through which meaning is conveyed. With depictive signals communicating meaning that is relatively complementary to the non-depictive speech they accompany, this half of the continuum largely coincides with the scope of the research program on co-speech gesture.

For cases closer to the right half of the continuum, relatively more information is communicated through depictive signals, and relatively less information comes from non-depictive speech. Embedded and independent depictions are located on this side of the continuum: Embedded depictions (more precisely, the stroke phase thereof, see subsection “Embedding”) convey meaning without the accompaniment of temporally co-occurring non-depictive speech, but are formally and functionally framed by the non-depictive speech surrounding the syntactic slot that they fill. Independent depictions also convey meaning without simultaneous non-depictive speech, and do so, according to Clark’s (2016) definition, independently of preceding or following speech. Without temporally overlapping non-depictive speech, depictive signals on this half of the continuum often contribute to the discourse essential information that is absent in the adjacent speech. In the sense that these are cases where depictive signals fill in temporal slots in the discourse, they are, in more general terms, cases of iconic gesture without co-occurring speech.

Thus conceptualized, the four depiction types as defined by Clark, of which the prototypical cases can be located as four points along the continuum, really capture the different levels of “division of labor” between non-depictive speech and depictive signals — or more generally speaking, between speech and depictions. In some cases, speech takes up more of the “load” of meaning communication; in others, the depiction “takes over,” showing meaning in iconically motivated ways. Importantly, the reconceptualization is not meant as a solution to the issues of the original typology. As the choice of term “continuum” suggests, it presupposes gradience rather than categoriality. Given the challenge of quantifying the amount of information communicated through speech as compared to depictions, the continuum is not one with strictly defined criteria either. Rather, it serves as a heuristic for identifying the varying levels of “trade-off” in terms of meaning contribution between non-depictive speech and depictive signals — not as dichotomous oppositions, but as two of the many sets of communicative resources available to the speaker in language use. It shows how, in staging different types of depictions, the speaker “packages” information in different ways, “distributing” it over speech and depictions, be they co-expressive, with or without “redundancy.”

### An Imbalance in the Literature

In addition to providing an alternative vantage point from which to consider the speech-depiction relations in the four depiction types identified by Clark (2016), the reconceptualization of the typology bears further theoretical relevance. Among other things, it brings to the fore an imbalance in the literature between studies on iconic gestures with and without co-occurring speech.

Largely coinciding with the left half of the continuum, where speech plays a relatively dominant role, and where adjunct and indexed depictions are located, the topic of iconic co-speech gesture has been core to modern gesture studies, with an extended body of dedicated research. Some scholars, for instance, have explored how gestures complement or supplement the semantics of their co-occurring speech (see the pioneering research by McNeill, 1992; Kendon, 2004); others have investigated how gesture and co-occurring speech package meaning in different ways (“imagistic” versus “linguistic”), debating how the two processes relate to each other (e.g., de Ruiter, 2000; Kita, 2000; McNeill and Duncan, 2000); still others have investigated the “deeper” link between gesture and co-occurring speech, as well as its implications in psychology and evolution (e.g., Stokoe, 2001; Arbib, 2005; Tomasello, 2008; McNeill, 2013a; Kita et al., 2017). Despite the late revival of the topic, formidable groundwork has been laid for the understanding of the workings of iconic co-speech gesture.

In contrast, phenomena that fall closer to the other end of the continuum — cases where iconic gesture communicates meaning without co-occurring speech, such as embedded and independent depictions — have not received equal attention. McNeill (2005, p. 5), for instance, identifies gestures that “occupy a grammatical slot in a sentence” as “speech-framed” or “speech-linked” gestures on Kendon’s Continuum (see also Kendon, 1988a; McNeill, 1992), but does not include them in further discussion. This is echoed by the general trend in gesture studies. Iconic representational gestures, for instance, have been explored by many, but with most of the studies focusing primarily on those co-occurring with speech (e.g., Müller, 1998a; McNeill, 1992; Kendon, 2004; Cienki and Müller, 2008; Enfield, 2009; Streeck, 2009). Fricke (2012, 2013) in her research delves into what she calls multimodal attribution, where gestures provide supplementary and sometimes essential information, but with the presence of co-occurring speech (see also Bressem, 2014). Similarly, Mittelberg and Evola (2014, p. 1734) observe that “iconic gestures can be produced to fill a semantic gap in speech, especially when representing spatial imagery like size, shape, motion, or other schematic, partial images which take advantage of the affordances of gestures versus speech,” but keep their focus on gesture-speech co-occurrence. Indeed, as Fricke points out, research in gesture studies has not yet moved beyond “the *assumption* that […] gestures can fill syntactic gaps in linear verbal constituent structures” (Fricke, 2013, p. 748; emphasis ours). As the continuum in Figure 5 shows, however, to focus only on gestures with co-occurring speech is to miss out on the other half of the picture.

To date, only a relatively small number of researchers have tapped into iconic gesture without co-occurring speech in naturalistic language use (but see reports from experimental settings, e.g., Sambre et al., 2019). Fricke (2012, cited in Müller et al., 2013, p. 65), for instance, identifies two types of gesture-speech integration, arguing that “gestures may be integrated by positioning, that is either through occupying a syntactic gap or through temporal overlap; or they might be integrated cataphorically, that is by using deictic expressions.” Though proposed for gestures in general, this distinction shares commonalities with Clark’s typology of depictions: Indexed depictions would be instantiations of cataphoric integration; the first kind of integration by positioning (“through occupying a syntactic gap”) would cover embedded and independent depictions; the second kind of integration by positioning (“through temporal overlap”) would include adjunct depictions.

Ladewig (2020) goes a step further and looks into “interrupted utterances,” that is utterances with an empty slot at the utterance-final position. With experiments, she shows that manual gestures can, much like canonical verbal constituents, be used to fill the empty slots in interrupted utterances and become an integrated part thereof, both syntactically and semantically. Coming from a different tradition but equally notable is the research conducted by Keevallik (2010, 2015, 2017, 2018, 2020), who systematically explores “bodily quoting,” a phenomenon in the context of dance instruction where the instructor employs bodily movements where a verbal quotation would normally be, in order to demonstrate the contrast between correct and incorrect performances to the students. Focusing on sequential temporality and drawing on data of multimodal interaction in multiple languages, she further demonstrates how verbal elements and bodily actions are mutually adapted in real time to create emergent multimodal patterns.

Analogous findings have also been reported from interactions in communicative ecologies other than those between hearing speakers of spoken languages. In the field of sign linguistics, for instance, Ferrara, Hodge, and Johnston have observed that enactments can be sequentially integrated into Auslan (Australian Sign Language), where the enactments can function in place of fully lexicalized manual signs, filling syntactic gaps as well as inferring or expressing semantic relations (Ferrara and Johnston, 2014; Hodge and Johnston, 2014). Based on her fieldwork on the alternate sign languages (see Kendon, 1988b) in the Arandic speaking communities of Central Australia, Green (2014) investigates how manual signs can be employed in discourse, with or without co-occurring speech, depending on the social protocols applicable to the current discourse. Specifically, she shows that signs can, in the absence of simultaneous speech, replace spoken lexical items in utterances, in some instances creating multimodal composite utterances with semantic contributions from both speech and sign (see also Green and Wilkins, 2014).

Finally, recent years have seen attempts at incorporating gesture into the theoretical framework of linguistic analysis, coming from various theoretical orientations and with different approaches [e.g., “integrated message model” (Bavelas and Chovil, 2000); “composite signal” (Clark, 1996); “composite utterance” (Enfield, 2009); “multimodal grammar” (Fricke, 2012); multimodal negation (Harrison, 2018); incorporation of gesture into Cognitive Grammar (Kok and Cienki, 2016); “mixed syntax” (Slama-Cazacu, 1976)]. Construction Grammar, in particular, sees a recent debate on Multimodal Construction Grammar (e.g., Steen and Turner, 2013; Schoonjans et al., 2015; Cienki, 2017; Hoffmann, 2017; Schoonjans, 2017; Ziem, 2017; Zima and Bergs, 2017). Arguing for nonverbal signals being as integral to language as canonical speech, these studies touch upon cases of gestures without simultaneous speech, acknowledging their crucial role in language use, but the primary focus remains on gesture-speech co-occurrence.

Essentially, phenomena on the right half of the continuum exemplify prototypical cases of “marginalia,” which, as Dingemanse (2017b, p. 195) identifies, are “typologically unexceptional phenomena that many linguists think can be ignored without harm to linguistic inquiry” — though not rare, “linguistic practice assigns them to the margin by consensus.” The handful of existing studies above only provide a first glance at, or around, the largely overlooked domain that is iconic gestures without co-occurring speech, revealing how limited our current understanding still is. Indeed, while certain subgroups of such cases have been studied, there has yet to be a general, systematic survey of the phenomenon itself — one that delimits it, explores its relations to speech, and examines how such gestures contribute to the resulting multimodal discourse — not least in spoken language interactions. In the following, we take a first step in this direction, within Clark’s framework of depicting, as it offers a schematic perspective on iconic meaning communication in general.

## Speech-Embedded Nonverbal Depictions

Up to this point, we have been arguing for the relevance of the overlooked domain from the theoretical side, contextualizing it against relevant research. In this section, we turn our attention to the empirical side of the phenomenon, which we now zoom in and elaborate on as “speech-embedded nonverbal depictions.” In addition to a detailed delimitation of the phenomenon based on real-life examples from our corpus, we also present a preliminary sketch of the complexity exhibited by such depictions.

While “speech-embedded nonverbal depictions” is not an opaque term, in order to properly identify our target phenomenon in relation to existing studies bordering the overlooked domain in the literature, we further define such depictions in more technical terms, as

— depictions that are embedded in speech, but that are not depictions of non-depictive speech,

where depictions are understood in the sense defined in Clark’s (2016) staging theory. The following excerpts present prototypical cases of such depictions.

(9) Bob Newhart on getting feedback from the audience when performing in the rain: “This one umbrella starts to [*stacks right fist on top of left fist in center-center, as if holding an umbrella, lightly shaking both arms vertically*]^a^, starts to [*stacks right fist on top of left fist in center-center, as if holding an umbrella, lightly shaking both arms vertically*]^b^, starts to jiggle.”

— *Conan*

**FIGURE 6:**
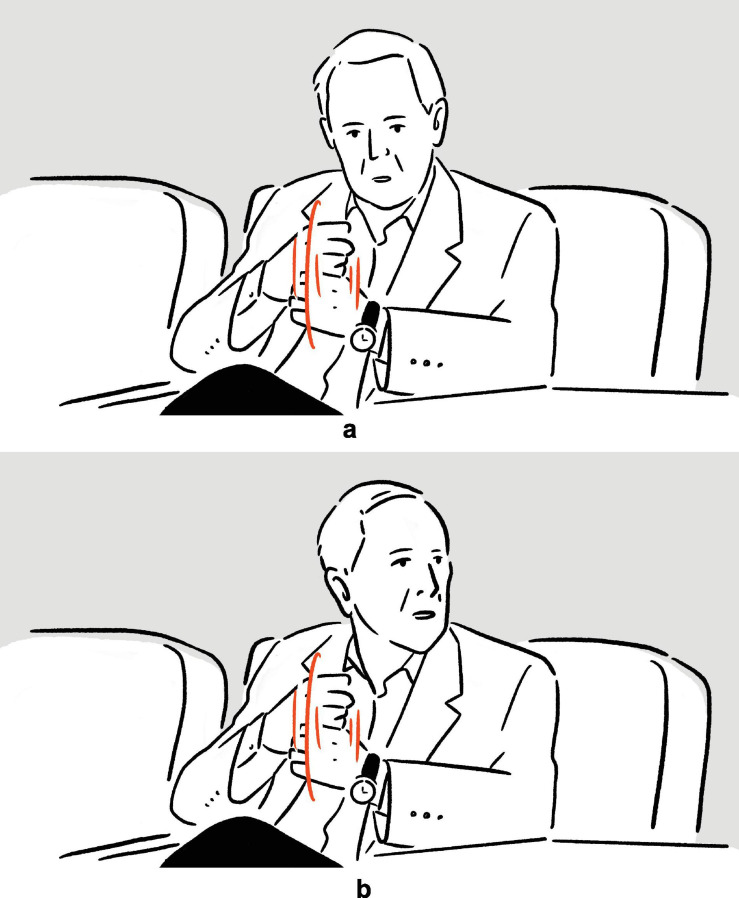
Depictions in (9).

(10) Zooey Deschanel on being refused priority boarding when traveling with her baby daughter: “and I was like, but [*moves both arms back and forth parallel to frontal plane, elbows bent, both palms up, left palm placed on top of right palm*]^a^. She needs to go on the plane.”

— *The Ellen DeGeneres Show*

**FIGURE 7:**
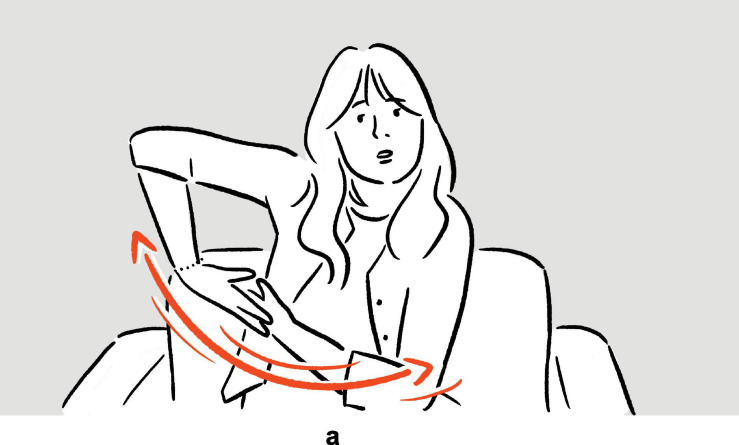
Depiction in (10).

In (9), Newhart recounts his experience of doing stand-up comedy in open air, where some of the audience were holding an umbrella because of the rain, and where, at some point, one umbrella started to jiggle because the person holding it was laughing. In the temporal “gaps” in his speech, he depicts, using mainly movements of the hands, arms, and shoulders, the jiggling of one of the umbrellas, thereby communicating the original scene of the event in an iconic way, with fine-grained motoric details. Sharing her experience of being denied priority boarding even though she was traveling with her baby daughter, Deschanel depicts in (10), after the word *but*, her reaction upon being so told, displaying actions typically associated with holding and rocking an infant, thereby enacting the scene, with imagistic details, to the audience of the talk show. In both (9) and (10), the nonverbal depictions are embedded in speech, filling the temporal “gap” therein. Employed to communicate meaning without the support of temporally overlapping speech, they exemplify the canonical use of speech-embedded nonverbal depictions in interaction.

It needs to be reiterated that speech itself can be depictive, descriptive, and indicative, and that cases of depictive speech fall under the category of depiction as well. For instance, the words in (11), which directly precede (10), are depictive of words uttered in the past, therefore a depiction.

(11) Zooey Deschanel on being refused priority boarding when traveling with her baby daughter: “and they were like, no, like, the people who get on first pay a lot of money for this privilege.”

— *The Ellen DeGeneres Show*

Quoting the ground crew member who denied her priority boarding, Deschanel is effectively staging a depiction of a past event, except that the past event is one where descriptive speech is uttered.

Ubiquitous and complex in their own right, depictions of descriptive speech — that is, canonical quotations — have long intrigued linguists and have a rich and extensive literature (e.g., McGregor, 1997; Tannen, 2007; Vandelanotte, 2009; Buchstaller, 2014; Spronck and Nikitina, 2019; see also Hodge and Cormier, 2019 for discussion in relation to depicting). Though indeed frequently observed in our corpus, such tokens are not included in our analysis, where the aim is to draw attention to overlooked phenomena in the literature. While eventually consolidating depictions across all modalities and signaling methods would be optimal, at the current stage, excluding canonical quotations allows us to prioritize focus on depictions that have hitherto eluded the attention of researchers — that is, depictions that are really marginalia (Dingemanse, 2017b) in the literature. This is reflected in our technical definition of speech-embedded nonverbal depictions presented above, where depictions of non-depictive speech are excluded, allowing us to focus on the core cases of iconic meaning communication.

Importantly, the exclusion of descriptive and indicative speech does not rule out cases of depictive speech from our analysis. A broad concept itself, depictive speech subsumes a number of phenomena and has been given various labels, such as multimodal quotation, sound symbolism, interjection, onomatopoeia, and ideophone (see e.g., Kita, 1997; Dingemanse, 2013, 2015), many of which have only recently been picked up in the cognitive-functional linguistics literature. Not only do they call for fuller exploration, they are also curious from the perspective of depicting and multimodality, as creative multimodal strategies are usually needed to establish iconic mappings between depictive speech and its depicted scene. Indeed, building on Dingemanse’s (2013) study, Clark (2019) identifies ideophones as depictions in the verbal modality, distinguishing between “free” and “codified” depictions, which can be illustrated, respectively, by the following examples, taken from our corpus.

(12) Jennifer Garner on accidentally kayaking into a busy harbor: “There were like [*vocalizes* brrr *sound; moves both hands slowly from left to right, palms facing each other, fingers spread, distance between palms constant*]^a^, like big boats.”

— *The Tonight Show Starring Jimmy Fallon*

**FIGURE 8:**
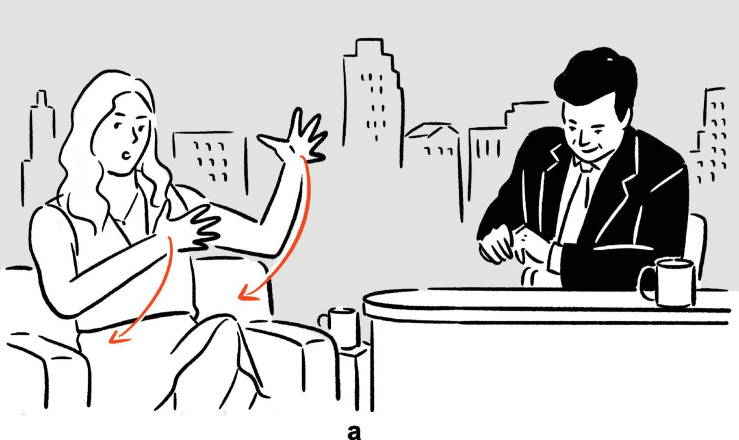
Depiction in (12).

(13) Chris Evans on bullying his brother (Scott Evans, seated to his left), in childhood: “And I just had the book, and just, [*vocalizes* whack, *moves left hand in a curve, from lower right periphery to upper left extreme periphery, close to where Scott’s head is*]^a^, and I hit him.”

— *Late Night with Seth Meyers*

**FIGURE 9:**
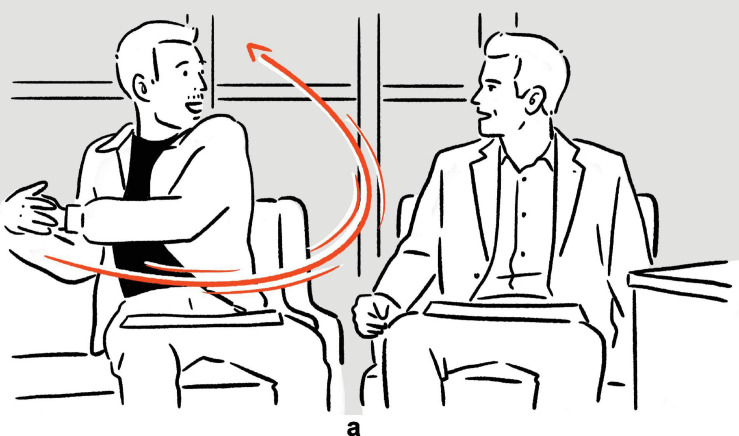
Depiction in (13).

In (12), where Garner recalls encountering big boats as she accidentally kayaked into a busy harbor, the big boats are referred to iconically. This is done, not just by her highly metonymic “bounding” (Streeck, 2008) manual gesture — where, drawing on the contiguity relation between her hands and the depicted object (Mittelberg and Waugh, 2014), the empty space between her hands is mapped onto some generic big boat — but also by the low-frequency *brrr* sound, depictive of the sound of boat horn. “Created *de novo*” (Clark, 2019, p. 235), *brrr* instantiates a free depiction. In (13), Chris Evans recounts hitting his brother with a thick book, depicting the scene by deploying a set of manual gestures, and, on top of that, *whack*, which is an ideophone codified in the English vocabulary for the sound of heavy strikes, and which is thus a codified depiction. Importantly, in both of the cases, the speaker establishes physical, specifically auditory, resemblance between the depictive speech and the depicted sound creatively, as it is humanly impossible to literally reproduce the latter.

It is following the definition spelled out in the present section, and with the modality-agnostic gesture phrase as the basic depiction unit, that the 217 tokens of speech-embedded nonverbal depictions were identified in our American TV talk show corpus. In what follows, we present further theoretical and methodological considerations — pertaining to the issue of embedding in particular — resulting from a closer examination of the 217 target tokens, as well as some observations regarding the internal complexities of the depictions.

### Embedding

In addition to distinguishing speech with different semiotic functions, another key notion that needs clear delimitation is embedding. It is a term that is particularly tricky because it can be understood either in terms of function or form, which are often conflated.

Clark (2016, pp. 325–326), in his typology, makes the functional distinction between embedded and independent depictions, with the former functioning as “parts of utterances” and the latter making “independent contributions to the discourse.” Empirically, this distinction is easily blurred. Consider the depiction in (14).

(14) Conan O’Brien: “How do you do that, do that again?” Kristin Chenoweth: “[*sings syllables* aye-ah *in high pitches*]”

— *Conan*

With the guest having just demonstrated some high-pitched singing, the host, impressed, asks the guest how that is done and requests that she do it again. In response to this, Chenoweth simply depicts her own singing, rather than verbally describe her singing technique. Contributing to the discourse without adjacent or co-occurring speech, Chenoweth’s depiction exemplifies what Clark (2016) identifies as an independent depiction.

Viewed on a more schematic level, the category boundary becomes less clear-cut. Among other things, the guest’s depiction only makes sense with the preceding discourse considered; it is co-dependent with the host’s question in carrying out their global function as a question-and-answer pair. As is the case for any signal in language use, the contributions made by independent depictions to the discourse are seldom, if ever, truly independent, a fact that undermines the functional basis of the embedded-independent distinction. In this sense, independent depictions are really as embedded as embedded depictions, except not on the level of the word or phrase, but on the level of the sequential organization of the interaction. Both types of depictions function as if they were verbal constituents, contributing meaning iconically without simultaneously co-occurring speech.

From a form-based perspective, embedding can be understood in temporal terms, that is the temporal overlap between a depiction and its adjacent speech. In discussing the temporal placement of depictions, Clark (2019, p. 241) points out that both embedded and independent depictions are ‘‘slotted’’ into utterances ‘‘without breaks or overlap,’’^12^ filling temporal gaps in utterances. That is, embedded and independent depictions do not differ in this regard. While there might be operationalizable ways to systematically untangle the overlap between embedded and independent depictions in function [see e.g., Lehmann’s (1988) gradient approach to clause linkage along multiple continua; and Hodge (2014) on clause-like units in signed language], they simply exhibit no difference in form as far as temporal overlap is concerned.

In accordance with our annotation, we adopt a form-based definition of embedding, in temporal terms, which effectively dissolves the categorical distinction between embedded and independent depictions in Clark’s typology, rendering both as instantiations of embedded depictions in our corpus. Specifically, we define an embedded depiction as one whose stroke phase does not overlap with temporally co-occurring speech --- as per our definition of the depiction unit, the stroke phase of a depiction is to be understood in the broad, modality-agnostic sense, as a schematization from the stroke phase of a manual gesture, and refers to the central, meaningful part of the movement of a depiction. In addition to allowing us to focus on the core component of depictions, this criterion also yields a more accurate picture of depiction embedding: As is the case for manual gestures, speakers in our corpus, in employing embedded depictions, are often observed preparing themselves ahead of the slot, timing the stroke of the depiction to be executed within the precise time frame of the slot.^13^

Reconsider example (13), repeated here as (15).

(15) Chris Evans on bullying his brother (Scott Evans, seated to his left), in childhood: “And I just had the book, and just, [*vocalizes* whack, *moves left hand in a curve, from lower right periphery to upper left extreme periphery, close to where Scott’s head is*], and I hit him.”

— *Late Night with Seth Meyers*

Recalling how he left a scar on the forehead of his brother by hitting him with a thick paperback book, Chris Evans stages a depiction after the second *just*, utilizing both his entire left arm and the codified ideophone *whack*. To demonstrate the full extent of the whacking, the speaker can be seen already retracting his left arm to his right at the second *just*, and retaining a gesture hold until after the word *him*. The gesture phrase therefore spans from *just* to after *him*. Despite the temporal overlap between speech and some of the depiction phases, we view the depiction in (15) as embedded, since its stroke is timed to fill the empty “slot” in the speech, in a sequential and not simultaneous manner, without temporal overlap.

Likewise, reconsider the jiggling example in (9), repeated here as (16).

(16) Bob Newhart on getting feedback from the audience when performing in the rain: “This one umbrella starts to [*stacks right fist on top of left fist in center-center, as if holding an umbrella, lightly shaking both arms vertically*], starts to [*stacks right fist on top of left fist in center-center, as if holding an umbrella, lightly shaking both arms vertically*], starts to jiggle.”

— *Conan*

Following the segmentation established above, this excerpt contains two depiction phrases, therefore two tokens of depictions. In addition to the preparation before the first depiction and the hold after the second, a “depiction hold” is also observed between the two depictions. In fact, all of the words included in the excerpt overlap temporally with some depiction phase. The two depictions are nevertheless embedded depictions, as their stroke phase does not temporally coincide with speech, but takes up a temporal gap in the sequence of the embedding speech.

In addition to preventing form-function conflation, defining embedding in temporal terms also helps to avoid some of the problems resulting from underspecification, such as those enumerated about (8), which lies on the boundaries of adjunct, embedded, and independent depictions in Clark’s typology. It is repeated here as (17).

(17) Tracy Morgan on the quality of his facial muscles: “Yeah, I’m your rubber-band man, [*vocalizes* brbrbrbr *sound, shakes head sideways quickly, causing facial muscles to vibrate accordingly*].”

— Conan

Despite the functional affiliation between the depiction and *rubber-band man*, since the stroke of the depiction takes place only after *man*, it is annotated as an embedded depiction in our corpus. Indeed, while there is no definitive way of determining whether the depiction functions more like an adjunct or a separate utterance, it is objectively, temporally embedded in the discourse.

One final implication of defining embedding in temporal terms pertains to the intersection between depictions and verbal indices. As mentioned, Clark’s (2016) definition of indexed depictions — as those that are indexed by indexical expressions in speech, such as *this* and *there* — does not explicitly address the fact that there exist two distinct kinds of verbal indices. Consider the indexical devices in (18), where the host claims he is not quick-witted enough to be on a game show, and (19), where the guest demonstrates her peculiar way of nodding.

(18) Conan O’Brien on his lack of quick wit: “I don’t have that, quick, [*snaps fingers of left hand thrice*]^a^.”

— *Conan*

**FIGURE 10:**
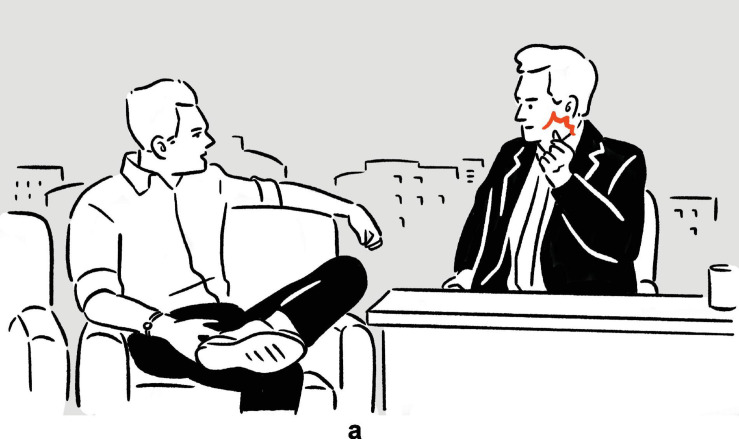
Depiction in (18).

(19) Emily Blunt on her impassive backchannels: “I just go like this [*nods head repetitively, quickly, but with little movement; maintains gaze at Seth Meyers, seated to her left*]^a^.”

— *Late Night with Seth Meyers*

**FIGURE 11:**
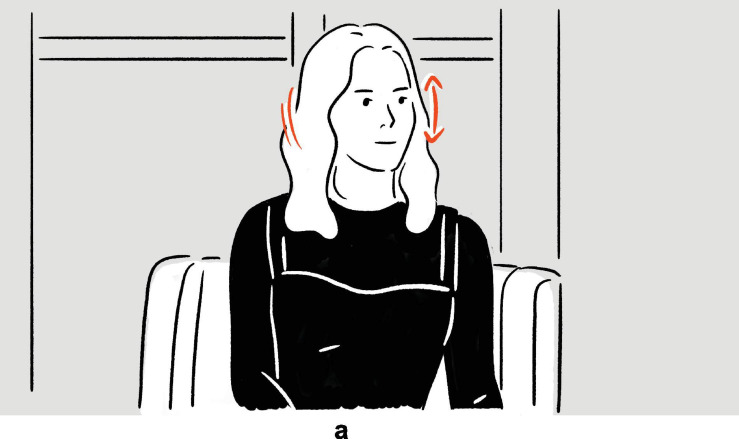
Depiction in (19).

Although verbal indices are present in both excerpts, they function in distinct manners. In (18) — where Conan depicts quick wit with finger snapping, which is associated with moments of epiphany, and therefore, metonymically, with people adept at witty comebacks — the verbal demonstrative *that* modifies what follows it, making it an indexical modifier. Accordingly, it is not that *that* indexes *quick, [*snaps fingers*]*, but that *that, quick, [*snaps fingers*]*, as a whole, indexes the kind of quick wit the host is referring to. In contrast, the indexical *this* in (19) — where Blunt simply depicts her own nodding — is a pronoun with indexical functions. As such, *this* by itself directly indexes the speaker’s peculiar head nod. In other words, where a depiction is employed in connection to an indexical modifier, what is indexed is not the depiction; where an indexical pronoun is used in conjunction with a depiction, it is the depiction that is indexed. Consequently, if indexed depictions are those that are indexed by indexical speech, they should only include cases of indexical pronouns, as in (19), and not cases of indexical modifiers, such as (18) — since in the latter, it is not the depiction, but the combination of the verbal index and the depiction, that is indexical.

Importantly, while the distinction between indexical modifiers and pronouns is crucial, the relations between verbal indices and depictions — specifically whether a depiction is indexed by a verbal index — are a functional concern. In other words, indexation is an issue on a dimension independent of our form-based definition of temporal embedding: A depiction can be indexed, embedded, neither, or both. Since both of the depictions in (18) and (19) occupy a temporal gap in speech without their stroke overlapping with speech, they are categorized as embedded depictions in our corpus, regardless of the fact that one of them is also verbally indexed and the other not. Though crucial to the understanding of depictions in general, a full-fledged exploration of the relations between depictions and verbal indices is left for further research.

### Complexities of Speech-Embedded Nonverbal Depictions

In addition to the issues concerning embedding alone, the tokens of speech-embedded nonverbal depictions in our corpus also exhibit complexities in other regards, of which we now offer a brief sketch. While the primary aim of the present paper is to draw attention to the phenomenon of speech-embedded nonverbal depictions, rather than to present an in-depth analysis, the following offers a glimpse into their theoretical and empirical potential, which further underscores the need for research on this overlooked topic.

Consider the depictions in (20), where Mulaney recalls stumbling wearing high heels, and where the depiction is preceded by *like*.

(20) John Mulaney on walking on heels: “It’s like, it was like, [*stretches out both arms sideways, tilts torso in different directions, as if trying to find balance*]^a^, when a, when a cow’s born.”

— *Late Night with Seth Meyers*

**FIGURE 12:**
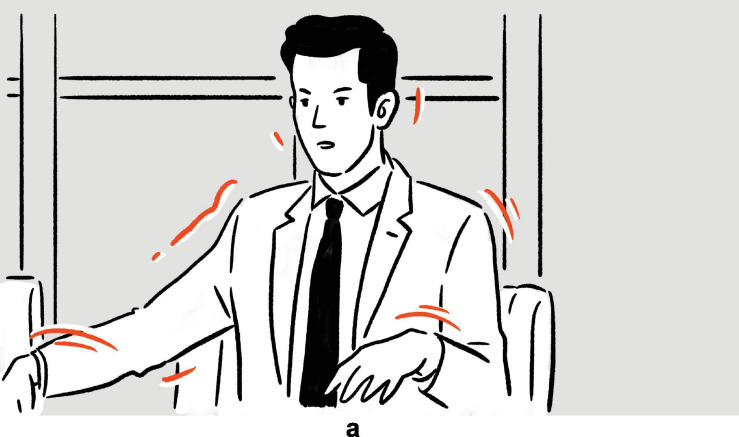
Depiction in (20).

Perhaps unsurprisingly, tokens preceded by *like* are frequently observed (see Golato, 2000; Streeck, 2002), due to *like*’s function as a quotative (see e.g., Tagliamonte and Hudson, 1999; Macaulay, 2001; Vandelanotte and Davidse, 2009), which indexes many of the functions depictions serve, such as quotation, enactment, demonstration, and pantomime (see Hodge and Cormier, 2019). What makes things less straightforward, however, is that *like* also often functions as a marker signaling hesitation or hedging, among many other things (see Miller and Weinert, 1995; D’Arcy, 2017). It further complicates the picture that, in cases like (20), the depiction is sometimes followed by verbal elements whose meaning overlaps with that of the depiction.^14^

With the stumbling depiction preceding *when a cow’s born*, it is plausible the depiction in (20) is the physical manifestation of the speaker’s thought process, specifically the mental simulation of the action he is trying to verbalize, which eventually results in *when a cow’s born* (e.g., de Ruiter, 2000; Hostetter and Alibali, 2008; see also Streeck, 2009 on “ceiving”). At the same time, it is also not unreasonable to suspect the depiction serves as a filler, one that fills the uncomfortable pause resulting from the speaker’s word search (see Goodwin and Goodwin, 1986; Gullberg, 1998; Hadar and Butterworth, 1997; Navarretta, 2015), before the speaker is able to “find their words.”

However, we also repeatedly come across cases like (21).

(21) Tina Fey on doing serious choreography: ‘‘If I had to be on Dancing with the Stars, I would be so shark-eyes,^15^ I would be like [*gazes at the front, into the distance; moves both arms in parallel, elbows bent, as if rowing a boat*]^a^, I would so panic all the time.”

— *The Ellen DeGeneres Show*

**FIGURE 13:**
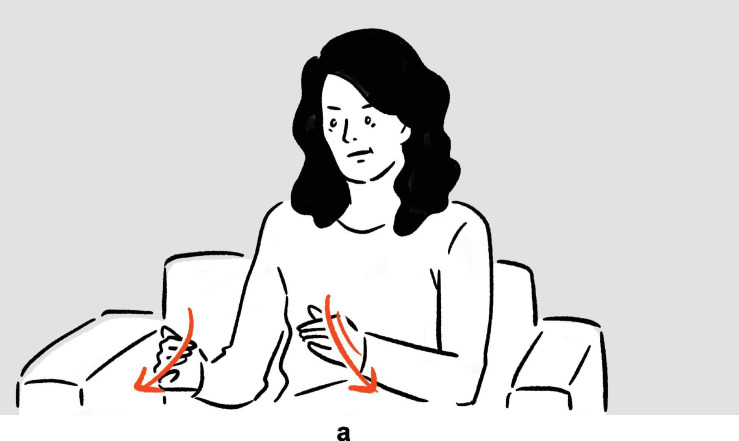
Depiction in (21).

Here Fey depicts how awkward and uncoordinated her movements would be if she were ever to go on a dance show. Like (20), the depiction in (21) is also preceded by *like*. Unlike (20), the depiction in (21) does not precede, but rather follows, *shark-eyes*, the verbal element whose meaning is similar to that of the depiction. In other words, the speaker first communicates the meaning verbally, saying *shark-eyes*, before staging a nonverbal depiction with highly similar semantics. The fact that the speaker first communicates her idea verbally, and then still proceeds to stage a depiction that is semantically “repetitive” — with the identical speech frame of *I would be* no less — shows that such nonverbal depictions cannot be conveniently dismissed as word-search fillers.

Indeed, cases of “multimodal iteration” (Hsu et al., to appear; cf. Johnston, 1996 on the “spiral” manner in which signing can unfold in Auslan), that is the phenomenon where the speaker communicates meaning in multiple combinations of modality and signaling method — specifically, in (20) and (21), verbal description and gestural depiction — may point to nonverbal depictions having different communicative potentials than descriptive speech (see Mittelberg, 2014 on “mediality effects”). In addition to exhibiting cross-modal dialogic resonance (see Du Bois, 2014), such tokens also showcase the reciprocal framing across modalities, whereby verbal and nonverbal elements profile certain aspects of one another (Kendon, 2004; Ferrara and Hodge, 2018). The mechanisms at work here may in turn contribute to the long-lasting debate whether gesture and speech are two separate processes, or manifestations of one single process (e.g., McNeill, 1992, 2013b; de Ruiter, 2000; Kita, 2000; Kendon, 2004), further adding to the reasons why speech-embedded nonverbal depictions deserve more attention.

Also strengthening the case for speech-embedded nonverbal depictions is the fact that they are observed embedded across different syntactic levels, from the level of the word (e.g., Example 9), phrase (e.g., Example 12), clause (e.g., Example 20), all the way to the level of the discourse (e.g., Example 14). The following depictions further exemplify this versatility.

(22) Lil Rel Howery on texting without looking at the screen: “People are just that good where they can just [*gazes at the front, into the distance; places both fists above lap, at lower center, flipping both thumbs up and down quickly, as if typing on a phone*]^a^.”

— *The Tonight Show Starring Jimmy Fallon*

**FIGURE 14:**
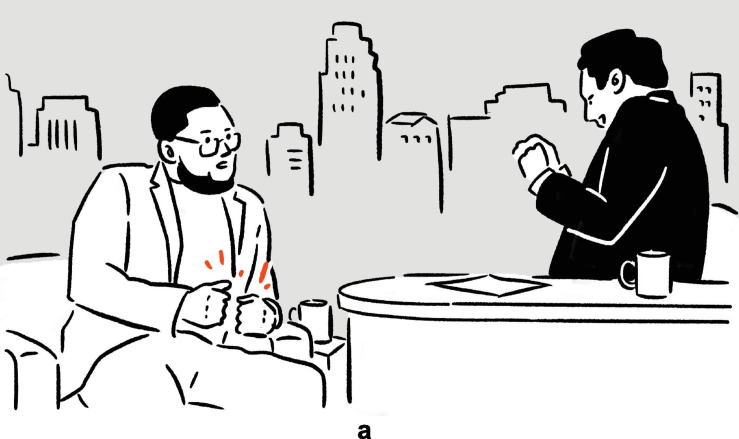
Depiction in (22).

(23) Cardi B on being mischievous as a kid: “I was like, ok I know, [*points with right index finger stretched, fingertip moving from center-center to right extreme periphery along a straight line*]^a^, go to the principal’s office.”

— *The Ellen DeGeneres Show*

**FIGURE 15:**
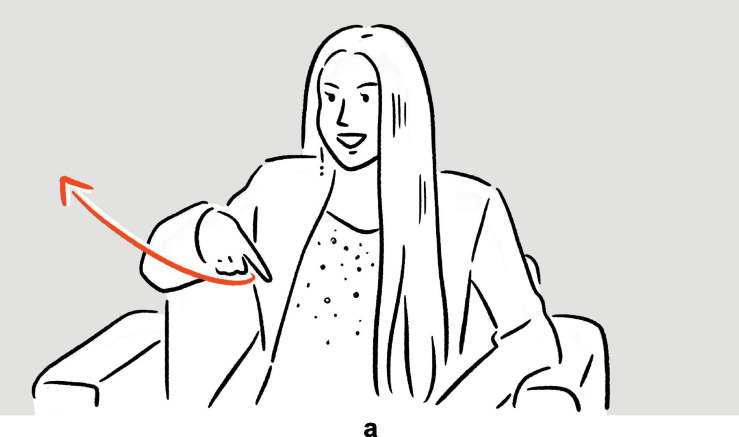
Depiction in (23).

In (22), Howery expresses his frustration with people who type on their phone without looking at the screen. In this case, the depiction is embedded on the level where a complex verbal phrase would otherwise be embedded. In (23), Cardi B stages how, after some mischief, her teacher asked her to go to the principal’s office. Here the embedding takes place on the level of the sentence, the depiction functioning like an imperative sentence otherwise would.

Though further research is needed, the versatility in syntagmatic depiction-speech integration already suggests the capability of nonverbal signals in “substituting” for structurally diverse verbal constituents, both in form and function. This echoes Ladewig’s (2020) recent findings, potentially also lending support to the view that nonverbal depictions as form-function pairings are not unlike verbal constituents — at least in the sense of Construction Grammar (Croft, 2001) and Cognitive Grammar (Langacker, 2008). This, of course, warrants a separate discussion that is beyond the scope of the present paper (see Ferrara, 2012; Hodge, 2014; Kok and Cienki, 2016; Wilcox and Occhino, 2016; Ruth-Hirrel and Wilcox, 2018; Ladewig, 2020).

The complexity and full potential of speech-embedded nonverbal depictions are also evident paradigmatically. For instance, reconsider once again the depictions in (9), repeated here as (24).

(24) Bob Newhart on getting feedback from the audience when performing in the rain: “This one umbrella starts to [*stacks right fist on top of left fist in center-center, as if holding an umbrella, lightly shaking both arms vertically*], starts to [*stacks right fist on top of left fist in center-center, as if holding an umbrella, lightly shaking both arms vertically*], starts to jiggle.”

— *Conan*

Here Newhart says an umbrella starts to jiggle, but what he depicts in the two embedded depictions is in fact not the jiggling of the umbrella per se, but the cause of the jiggling, namely the person laughing whilst holding the umbrella, who is in turn represented by Newhart’s fists. Despite the “mismatch,” Newhart is able to get his message across because of the metonymic relations that are at play here: part for whole (the fists for the umbrella holder), and cause for effect (the umbrella holder’s action for the umbrella’s movement). Furthermore, the phrase *this one umbrella starts to jiggle* (whether the notion of jiggle is communicated through the depiction or the word *jiggle*) is itself a metonymic way of saying a member of the audience starts to laugh (effect for cause: the umbrella’s movement for the person’s action).

Paradigmatic complexities are also manifest in the observation that speech-embedded nonverbal depictions are sometimes employed back to back, such as in (25), in which the host demonstrates how he would not be able to refrain from actually eating if he were to play a role that requires eating on scene.

(25) Conan O’Brien on being unable to refrain from savoring the food if required to eat on scene: “I’d be, even in a drama, they’d be like, Conan’s the murderer, [*vocalizes* kahm-ahm, *moves mouth as if biting and chewing; moves hands in parallel, from lower center toward upper center near own mouth, fingers touching on both hands, as if holding a hamburger*]^a^ — [*vocalizes* hum-um, *sucks own fingers*]^b^ — [*stretches out right index finger in upper right periphery, as if signaling some imaginary addressee to wait until he is done eating; moves mouth as if chewing*]^c^.”

— *Conan*

**FIGURE 16:**
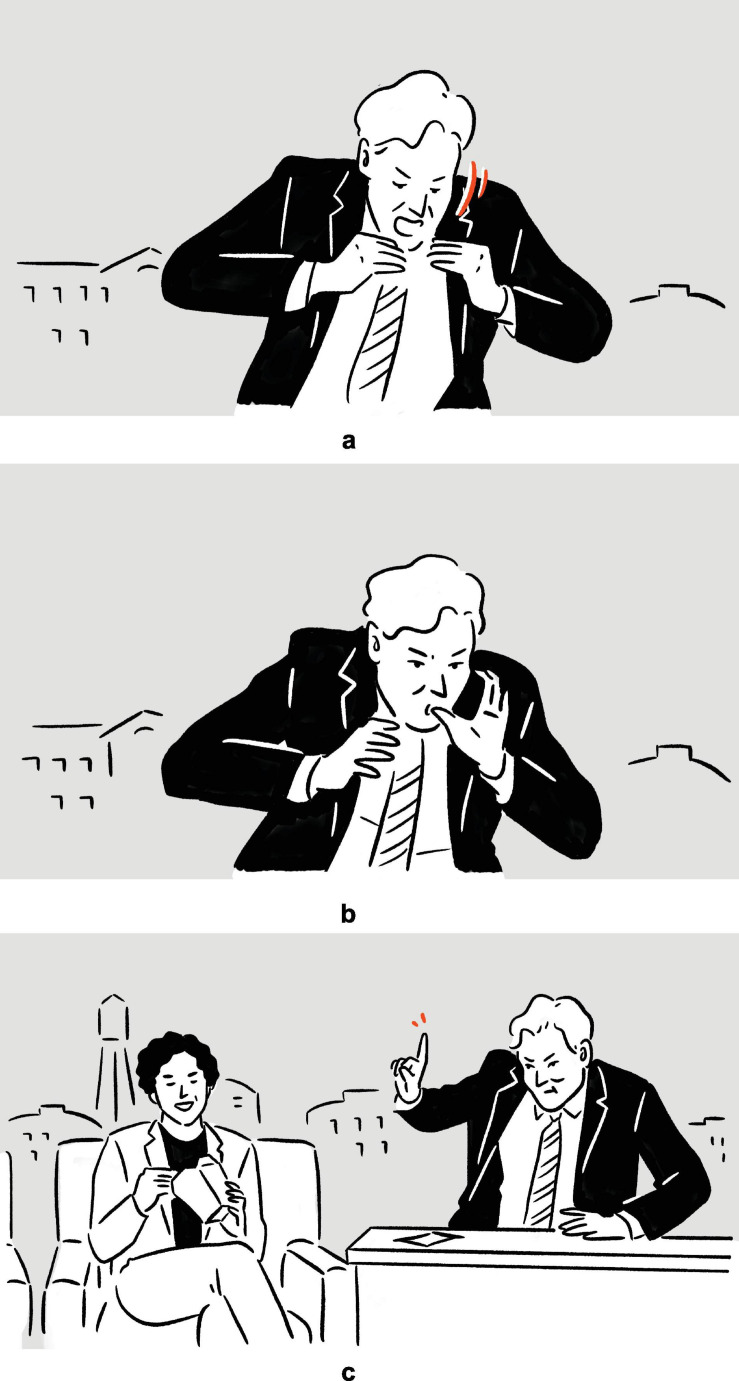
Depictions in (25).

In the first set of actions, Conan depicts himself ferociously munching on some burger type of food item; the second depiction includes the finger sucking action typically associated with someone enjoying fast food; in the third and final set of actions, Conan depicts how he would prioritize actually eating over acting. Notably, in all three of the depictions, which are staged consecutively without speech “intervening,” Conan can be observed maintaining the same bodily posture, consisting primarily of raised shoulders and upper arms.

What really sets this example apart is the fact that the understanding of the later depictions hinges on the understanding of the earlier ones. Without the first depiction, the finger-sucking gesture in the second depiction would be a lot harder to make sense of. Likewise, were the first two depictions absent, the third depiction would hardly be decipherable on its own. In other words, the later depictions build and elaborate on prior depictions, along the same storyline. Together, the co-dependent depictions, bound together by the common thread that is Conan’s sustained posture, contribute to a composite structure with a complex meaning.

On a more theoretical level, complex composite depictions (Hsu, 2019) like (25) are significant to the discussion on the role of nonverbal signals in language, and therefore also to the above-mentioned Multimodal Construction Grammar debate, as they demonstrate that even singular actions (Müller, 2010, cited in Ladewig, 2014) — that is, actions created and assembled *ad hoc* (see Brône and Zima, 2014), for highly local purposes — can be combined to create larger structures, undermining the argument that gestures are not “linguistic” simply because of their lack of recurrence and low frequencies (see Schoonjans, 2017). The observation that the component depictions in the composite series share a common posture as their “base” (Hsu, 2019), also echoes comparable phenomena that have been identified in the literature, such as “locution cluster” (Kendon, 1972), “catchment” (McNeill, 2005), and “frame hold” (Sowa, 2006).

Cases of composite depictions can be further complicated by viewpoint changes. Consider again the depiction sequence in (6), repeated here as (26).

(26) Lauren Ambrose on backstage costume change on Broadway: “I mean sometimes it’s like twenty seconds, for like, full-on, [*vocalizes whistle-like* fsss *sound, moves both hands vertically, fingers spread, in opposite directions, in front of head and torso*] — [*vocalizes whistle-like* ffft *sound, gazes at the front, into the distance, moves both hands along sagittal axis away from body, fingers spread, palms away from body*].”

— *Late Night with Seth Meyers*

Similar to (25), the two depictions in (26) depict two subevents unfolding in sequence which are part of a larger event: The backstage staff on Broadway first changed Ambrose’s makeup and costume, and, after that, pushed her back to the stage. In addition to the composite structure, a striking viewpoint shift is observed between the two depictions. In the first depiction, the speaker takes on her own viewpoint in the depicted event (one can also argue that, since her hands depict the staff members’ hands, she also takes on the staff members’ perspective simultaneously; see e.g., McNeill, 1992; Parrill, 2009; Dancygier and Sweetser, 2012 on dual viewpoint). In the second, she takes on the perspective of the backstage staff member who pushed her back to the stage. Remarkably, the only overt cue signaling this shift in perspective is her gaze behavior: During the first depiction, the speaker appears to be looking at the host; during the second, her gaze is averted, focused instead on something in the distance. Tokens such as this echo findings in recent studies (e.g., Sidnell, 2006; Sweetser and Stec, 2016), which situate speech-embedded nonverbal depictions at the intersection between gesture, viewpoint, and gaze (see also Stec et al., 2016; Janzen, 2017).

The communicative potential of speech-embedded nonverbal depictions can also be exploited jointly across multiple speakers, as is the case in (27), an extended excerpt of which (13) is a part. As Chris Evans recounts hitting Scott Evans, his brother, with a thick book, Scott, seated to Chris’s left, joins in the storytelling, using not words, but depictions.^16^

(27) Chris Evans (A) on bullying his brother, Scott Evans (B), who is seated to his left, in childhood:
  A: And I just had the book,
  and just, ^∗^[*D1*]^∗^, and I hit him,
  B: ^∗^[*D2*]^∗^
  A: and as ^∗^soon as I^∗^ hit him, [*D4*]
  B: ^∗^[*D3*] ^∗^
    D1: Vocalizes *whack*; moves left hand in a curve, from lower right periphery to upper left extreme periphery, close to where B’s head is.
    D2: Tilts head away from A.
    D3: Traces scar on left forehead with left index.
    D4: Vocalizes *brrr*; touches forehead with fingertips of left hand, fingers touching, moves left hand toward upper left extreme periphery, spreading fingers along movement.

— *Late Night with Seth Meyers*

**FIGURE 17:**
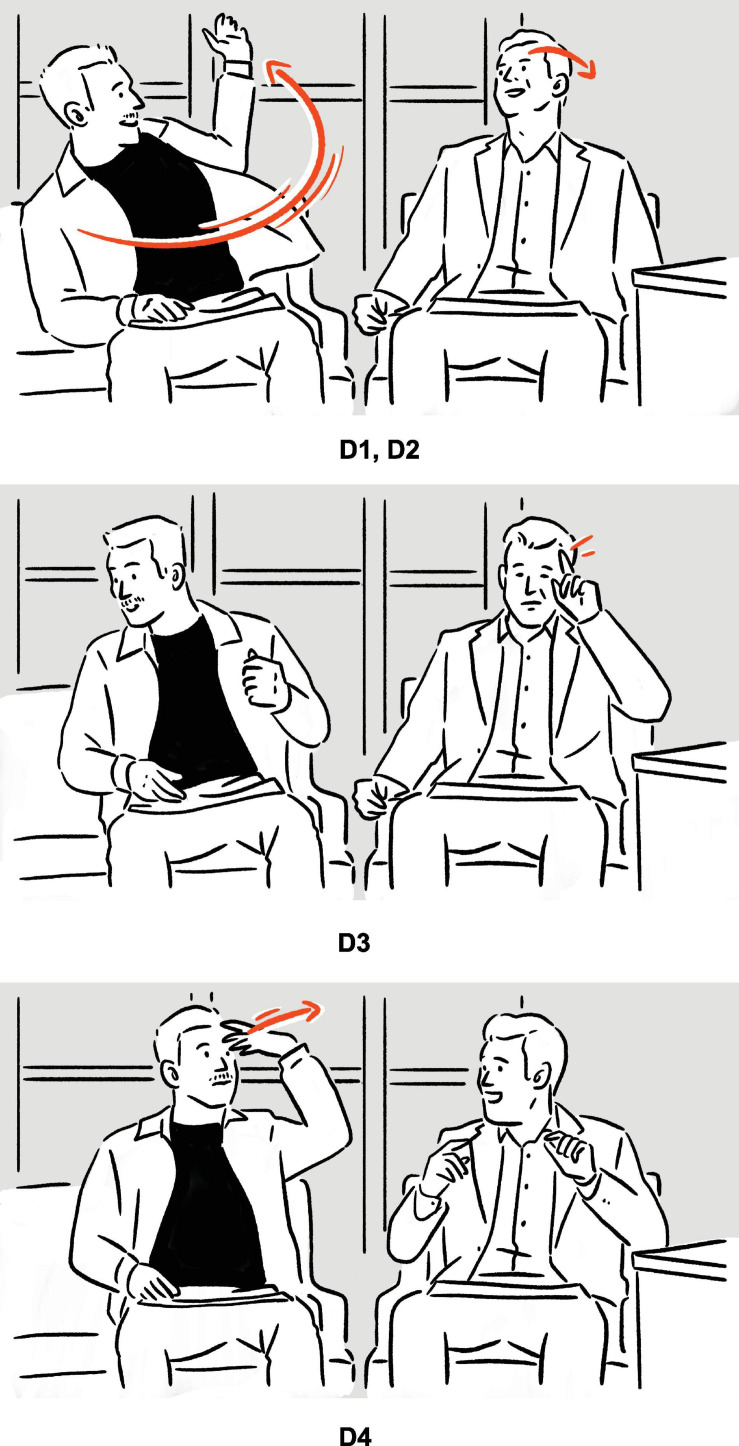
Depictions in (27).

Almost as soon as Chris stages the *whack* depiction, Scott is seen staging the second depiction, which is an enactment of his response to being hit by Chris. When Chris is at *soon as I*, Scott again contributes to the story, depicting the scar by locating and finger-tracing its shape on his forehead, before Chris stages the fourth depiction, which demonstrates, in an exaggerated manner, the spurting of the blood that came out of Scott’s forehead. The series of depictions, from both parties, goes on beyond the excerpt. As in (25) and (26), the depictions are co-dependent in meaning. Unlike in (25) and (26), the depictions in (27) are not all staged by one single speaker, but are staged jointly by two speakers, with causation between the depictions, bringing in the complexities of an additional, interactional dimension to the analysis.

The above is a very brief sketch of some of the complexities of speech-embedded nonverbal depictions, based only on tokens taken from our American TV talk show corpus, where the annotated data still await in-depth analysis. The rich and challenging cases this alone has already provided us with, nonetheless hint at the fact that speech-embedded depictions are not merely theoretically significant, but abundant in curiosities of language use and interaction as well.

## Concluding Remarks

Drawn to nonverbal iconic language use, and informed by Clark’s recent account of depicting in everyday interaction, we turned to video recordings of American TV talk shows, a context rich in depictions. The annotation of the data proved less straightforward than expected, an issue that underlines our limited understanding of this domain of research. In addition to operationalizing relevant theoretical notions, a critical reconsideration of depiction-speech relations, on the basis of Clark’s typology of depictions, was carried out, resulting in a gradient reconceptualization of depictions in terms of meaning contribution from non-depictive speech and depictive signals. This led to the identification of a largely overlooked domain — cases where meaning is communicated through iconic nonverbal signals, without temporally co-occurring speech — which we zoomed in on as “speech-embedded nonverbal depictions.” Taking into consideration existing literature as well as the variety of tokens in our corpus, we arrived at a carefully delimited definition of such depictions, in turn bringing to the fore numerous observations, many of which pertain to current discussions in cognitive linguistics, gesture studies, and multimodal communication.

As an initial step into the largely uncharted territory, it goes without saying the present study is limited in several ways. Among them is the type of data examined. The majority of the speakers in our corpus are professional actors or comedians, a fact that likely has an effect on the frequency, elaborateness, and spontaneity of the depictions they stage. Nonetheless, while true spontaneity is hardly attainable, it is undeniable that American TV talk shows, which are themselves a specialized context, contain unscripted elements. In addition, as the staging theory (Clark, 2016) suggests, performativeness is an inherent aspect of depicting (as has also been reported for Auslan; see Hodge and Ferrara, 2014), that is the signaling of meaning through showing. Dramatizations and exaggerations have also been reported to be common in narratives in general (see e.g., Bavelas et al., 2014; Stec et al., 2015). On a more schematic level, the present paper is focused primarily on spoken language interactions, due in part to the fact that the topic of the current study is particularly marginalized in spoken language linguistics. This is in contrast with signed language linguistics, which sees many relevant phenomena being more established topics in its literature (see among many others the above-mentioned Liddell, 2003; Wilcox and Occhino, 2016; Ferrara and Hodge, 2018). Future studies on the topic will benefit from larger datasets that are more diverse in terms of communicative ecologies (see Beukeleers and Hsu, 2019 for an initial attempt), which will also facilitate quantitative analysis, potentially bringing in insights from a different angle.

The scope of the data notwithstanding, the tokens in our corpus already shed light on some of the natural next steps for depiction researchers to embark on. Among them are depiction-speech relations, multimodal iteration, complex composite depictions, viewpoint in multimodal interaction, as well as jointly staged depictions and the causation therein. These are the tracks along which we are currently carrying out analysis of the tokens. Though not explicitly touched upon in the present paper, the tokens also point to a number of other directions in which future studies can proceed, such as issues pertaining to intersubjectivity, the performative aspect of depictions, depicting and language acquisition, and motivations for employing speech-embedded nonverbal depictions.

Speech-embedded nonverbal depictions are situated, not only at the crossroads of numerous research traditions, but also among intertwined modalities and signaling methods, which prove tricky to untangle. Nonetheless, as showcased by the versatile and complex ways in which speech-embedded nonverbal depictions are employed in real-life interaction, a full picture of language use will not be complete without a systematic account of such depictions.

## Data Availability Statement

The raw data supporting the conclusions of this article will be made available by the authors, without undue reservation.

## Author Contributions

H-CH conceptualized the study, compiled and annotated the data, carried out the analysis, and wrote the manuscript. GB and KF provided guidance in the conceptualization of the study, assisted in the analysis, and contributed to the revision of earlier versions of the manuscript. All authors contributed to the article and approved the submitted version.

## Conflict of Interest

The authors declare that the research was conducted in the absence of any commercial or financial relationships that could be construed as a potential conflict of interest.

## Figures and Tables

**APPENDIX:**
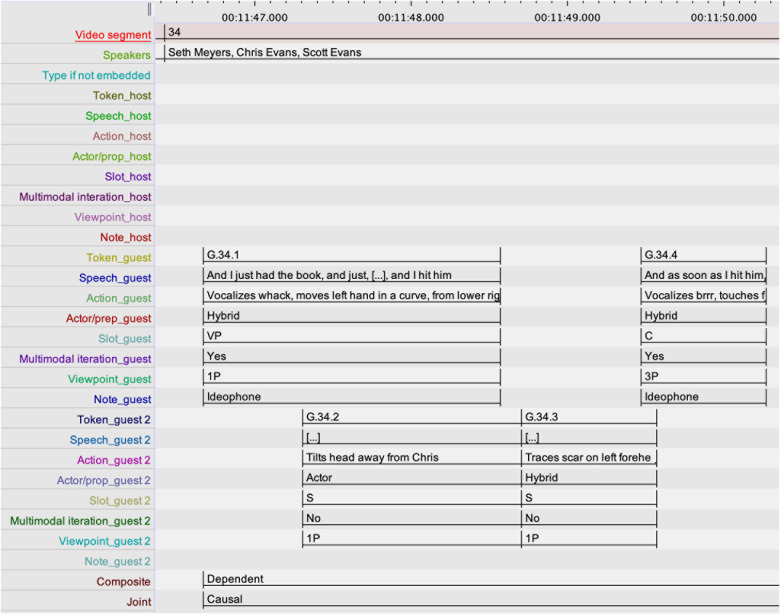
Screenshot of annotation in ELAN (of Example 27).

## Footnotes

1 In line with Clark’s (2016) notation, nonverbal signals are described in italics. Nonverbal signals in brackets do not co-occur with speech; those that do co-occur with speech are in parentheses, their co-occurring speech underlined. Where drawings are included, the superscript letters in the token description indicate the corresponding drawings.

2 Not all actions observed in the tokens are addressed in the present paper, for theoretical reasons (see section “Speech-Embedded Nonverbal Depictions”) as well as methodological ones: Cuoco, for instance, can be observed displaying some facial expression at the same time the depiction in (1) is being staged. While the facial expression may be depictive of her facial expression during (or, more likely, around) the time of the depicted event, it is equally plausible that it is a display of her stance toward the fact that she was tricked by her fiancé into doing something as scary as a canyon swing, a piece of information she has revealed in prior discourse. In this sense, the facial expression is, at best, peripheral to the depiction of the canyon swing, if part of any depiction at all. Significant in their own right (see e.g., Tabacaru, 2014; Janzen, 2017), such actions fall beyond the focus of the present paper that is prototypical depictions, therefore not included in the token description.

3 The drawings included in the present paper are made by Yuga Huang (yugagagahuang.myportfolio.com), based on screenshots taken from four TV talk shows (see section “Methods”): *The Ellen DeGeneres Show, Late Night with Seth Meyers, Conan*, and *The Tonight Show Starring Jimmy Fallon.*

4 As is the case for any depiction, the iconic relation between Cuoco’s actions and the canyon swing scene is a complicated one, involving not only physical resemblance between the articulators and the depicted scene, but also contextual information, the embodied encyclopedic knowledge of Cuoco and her audience, and, importantly, an array of complex metonymic mappings (see Arnheim, 1969; Mittelberg and Waugh, 2014). Given the focus of the present paper, the detailed mechanisms of metonymy in the examples are discussed concisely and selectively.

5 ELAN (Version 5.2) [Computer software]. (2018, April 04). Nijmegen: Max Planck Institute for Psycholinguistics. Retrieved from https://tla.mpi.nl/tools/tla-tools/elan/

6 The word *bingo* is used in this specific case as a conventionalized ideophone, therefore categorized not as descriptive speech, but as a depictive signal (see section “Speech-Embedded Nonverbal Depictions”).

7 Following the practice in Conversation Analysis, we use (.) to indicate a short pause or micro-pause, shorter than 500 ms (see Mondada, 2016).

8 With the speaker taking up the roles of multiple actors, these actions also instantiate what Clark (2016) identifies as an actor-actor hybrid depiction.

9 The *brbrbrbr* sound is an ideophone, albeit a non-conventional one. Depictive rather than descriptive (see section “Speech-Embedded Nonverbal Depictions”), it is a part of the depiction.

10 If the reader finds the Tracy Morgan quote confusing, that is because it is meant to be confusing, as evidenced by what the host, Conan O’Brien, remarks immediately after, “it’s the most sense you’ve made all night.”

11 Incidentally, Figure 5 bears resemblance to figures presented in Dingemanse (2017a) and Ferrara and Halvorsen (2017), which capture the continuum along which the dual semiotic properties — of being both depictive and descriptive — of ideophones and iconic lexical signs can be exploited. The resemblance is however only on the level of visualization: What Figure 5 presents is not the interplay between the different semiotic properties within individual signals, but how depictive signals (signals whose semiotic properties are predominantly depictive) relate to non-depictive speech in the four types of depictions identified by Clark (2016).

12 The criterion “without breaks or overlap” also proves problematic empirically. See discussion on temporal overlap immediately below.

13 Clark in his (2016, p. 340) paper touches upon what he calls “phases of discourse” and “discourse timing,” but does not explicitly elaborate on the different ways in which these notions relate to one another in the four depiction types he identifies.

14 The observations made in this paragraph about *like* are also largely applicable to *just*, as in (15) and (22).

15 If someone has shark eyes, it means the person’s gaze is empty and absent.

16 Given the complexity resulting from the temporal overlap between the two speakers, and informed by the conventions of Conversation Analysis, we employ asterisks (rather than brackets, which in the present paper already indicate nonverbal signals without co-occurring speech) to mark the beginning and end of simultaneous events (cf. Mondada, 2016). To facilitate readability, the depictions are dubbed “Dn” and described after the text excerpt.
